# The role of heterogeneity in asthma: a structure-to-function perspective

**DOI:** 10.1186/s40169-017-0159-0

**Published:** 2017-08-03

**Authors:** Justin K. Lui, Kenneth R. Lutchen

**Affiliations:** 1 Department of Medicine, University of Massachusetts Medical School, 55 Lake Avenue North, Worcester, MA 01655 USA; 2 Department of Biomedical Engineering, Boston University, Boston, MA 02215 USA

**Keywords:** Asthma, Heterogeneity, Ventilation, Lung mechanics, Computational modeling

## Abstract

A number of methods have evolved through the years in probing the dysfunction that impacts mechanics and ventilation in asthma. What has been consistently found is the notion of heterogeneity that is not only captured in the frequency dependence of lung mechanics measurements but also rendered on imaging as patchy diffuse areas of ventilation defects. The degree of heterogeneity has been linked to airway hyperresponsiveness, a hallmark feature of asthma. How these heterogeneous constriction patterns lead to functional impairment in asthma have only been recently explored using computational airway tree models. By synthesizing measurements of lung mechanics and advances in imaging, computational airway tree models serve as a powerful engine to accelerate our understanding of the physiologic changes that occur in asthma. This review will be focused on the current state of investigational work on the role of heterogeneity in asthma, specifically exploring the structural and functional relationships.

## Introduction

Asthma is an obstructive lung disease commonly associated with increased airway hyperresponsiveness (AHR) resulting in bronchoconstriction and airway inflammation. When provoked, the airways constrict in a heterogeneous pattern leading to airflow obstruction and impedance of gas transport to and from the lungs. The concept of heterogeneity extends past what occurs structurally to also characterizing both the clinical presentation (or phenotype) [1, 2] and underlying pathogenesis (or endotype) of the disease [3, 4]. Both clinical phenotypes and molecular endotypes are interrelated by genetic factors which continue to play a key role in the development of personalized approaches to the diagnosis and treatment of asthma [2, 5, 6]. Although beyond the scope of this review, studies have provided evidence in the existence of multiple genes associated with lung function in asthma that may lead to the degree of heterogeneous distribution of structural defects and consequent functional defects [7–10]. Our review focuses on the structure–function consequences of heterogeneous disease not the genetic origins of such. Specifically, we will focus primarily on the various techniques in assessing how heterogeneous changes in lung structure caused by asthma which result in changes to mechanical and ventilation function.

The degree of mechanical dysfunction can be assessed from measurements of flow (*Q*_*ao*_) and pressure (*P*_*ao*_) at the airway opening from which an in-phase (or energy loss) component or lung resistance (*R*_*L*_) and an out-of-phase (or energy storage) component or lung elastance (*E*_*L*_) of this relationship can be derived by what is known as the forced oscillation technique (FOT) [11–16]. Through evaluation of the respiratory system response to forced flow, the technique enables a rapid assessment in the degree of frequency dependence of *R*_*L*_ and *E*_*L*_ which allows inference on the severity of airflow obstruction [17–22] and the degree of bronchodilation after intervention [23–26]. While such methods provide evidence of degree in the heterogeneity of constriction, the ability to visualize ventilation abnormalities due to bronchoconstriction in asthma has been limited until recent advances in imaging. With the advent of hyperpolarized helium-3 magnetic resonance imaging (HP ^3^He MRI), there is now a noninvasive method to directly visualize areas of ventilated airspaces within the lung [27–36]. While normally ventilated lungs exhibit a homogeneous distribution of signal, obstructive lung diseases such as asthma demonstrate heterogeneously distributed non-ventilated areas known as ventilation defects. How these structural changes alter function has only been recently shown through computational modeling approaches of the lung [21, 22, 27, 37–41].

## Heterogeneity of lung mechanics

### The forced oscillation technique

First introduced in 1956 by Dubois et al. [13], the FOT is a noninvasive tool for the measurement of the mechanical impedance of the respiratory system. The technique involves delivery of pressure oscillations either around the chest wall inside an enclosed chamber (*P*_*cw*_) or alternatively at the mouth at the site of airway opening (*P*_*ao*_), which is more commonly used. The predefined oscillations were originally within the range of 4–32 Hz with a fixed limited magnitude to allow for spontaneous breathing. The apparatus generally consists of a loudspeaker to deliver the pressure signal and utilizes a high inertance bias tube placed in parallel to allow for a minimization of energy loss from the pressure oscillations while also providing fresh air to prevent carbon dioxide buildup within the dead space. By measuring flow at the airway opening (*Q*_*ao*_), the ratio of *P*_*cw*_ */Q*_*ao*_ can be used to calculate the transfer impedance of the system (*Z*_*tr*_). Alternatively, the ratio of *P*_*ao*_ */Q*_*ao*_ can be used to calculate the input impedance of the system (*Z*_*in*_)—this is often referred to as the impedance of the total respiratory system (*Z*_*rs*_) with contributions from both the lungs (*Z*_*L*_) and the chest wall (*Z*_*cw*_). The corresponding relationship is a complex ratio expressed as a function of oscillation frequency, in radians (*ω*), divided into an in-phase resistive component (*R*_*rs*_) and an out-of-phase reactive component (*X*_*rs*_):

1 $$Z_{rs} \left( \omega \right) = R_{rs} \left( \omega \right) + jX_{rs} \left( \omega \right)$$

in which *j* is defined as $\sqrt { - 1}$, an imaginary number. In its individualized components, *R*_*rs*_ embodies the dissipative mechanical properties or energy losses of the respiratory system over one cycle at a particular frequency, while *X*_*rs*_ embodies the energy storage capacity. To isolate the input impedance of the lungs alone (*Z*_*L*_), an esophageal balloon catheter is used to measure the intraesophageal pressure (*P*_*es*_) which approximates to be the intrapleural pressure (*P*_*ip*_) from which a transpulmonary pressure (*P*_*tp*_) can be calculated by the difference between *P*_*ao*_ and *P*_*es*_, given as:

2 $$Z_{L} (\omega ) = \frac{{P_{ao} (\omega ) - P_{es} (\omega )}}{{Q_{ao} (\omega )}}$$

Over the frequency range of 4–32 Hz, however, *R*_*rs*_ has been found to be relatively constant with frequency while *X*_*rs*_ has been found to increase monotonically with frequency [12, 14]. These data do not reflect much detail in the why or where or how they become abnormal in diseased states such as asthma. In contrast, lower frequency ranges (<10 Hz) have been found to be more relevant to mechanical properties with increased sensitivity to structural changes that capture phenomena such as airway wall distensibility [42, 43], tissue viscoelasticity [44, 45], smaller parallel time-constant heterogeneity [46], and expiratory flow limitation [47, 48]. Obtaining lung mechanics near spontaneous breathing frequencies becomes challenging as it requires subjects to remain apneic at functional residual capacity (FRC) for extended periods of time while forced oscillations are delivered [49]. A solution to this has been the design of a computer-driven optimal ventilation waveform (OVW) consisting of seven non-sum, non-difference sine waves with frequencies spanning from 0.156 to 8 Hz [18, 19, 50, 51]. The OVW would ventilate the subject with a normal tidal volume of air per cycle with a linear piston pump while simultaneously delivering forced oscillations at all the target frequencies [50]. The clinical utility of using FOT at lower frequencies is that such data is sensitive to changes in tissue viscoelastic properties as well as the occurrence and impact of heterogeneous airway constriction inclusive of capturing whether there are near-closures throughout the airway tree [18, 23]. To quantify these phenomena one can apply inverse modeling to the data, but to better understand how explicit structural changes in a whole lung might impact the data, one can apply forward modeling approaches.

## Model approaches to lung mechanics

### Inverse modeling

A simple inverse model used to characterize *Z*_*rs*_ relating *Q*_*ao*_ and *P*_*ao*_ of the respiratory system is the single compartment model comprising elements of resistance (*R*_*rs*_), elastance (*E*_*rs*_), and inertance (*I*_*rs*_) in a linear series described below:

3 $$Z_{rs} \left( \omega \right) = R_{rs} \left( \omega \right) + j\omega I_{rs} \left( \omega \right) + \frac{{E_{rs} (\omega )}}{j\omega }$$

*E*_*rs*_, dominant at low frequencies, represents the stiffness of parenchymal tissue and the chest wall. *I*_*rs*_, dominant at high frequencies, represents the energy required to move gas within the lungs in response to forced oscillations [12]. The resonant frequency, *ω*_*0*_, in which *X*_*rs*_ is zero, can be determined by rearranging Eqs. 1 and 3 to be given as:

4 $$\omega_{0} = \sqrt {\frac{{E_{rs} (\omega )}}{{I_{rs} (\omega )}}}$$

Over low frequencies in which *ω* ≪ *ω*_0_, the contribution of *I*_*rs*_ approaches zero, and the *X*_*rs*_ is essentially determined by *E*_*rs*_ which is related in common practice as:

5 $$E_{rs} \left( \omega \right) = - \omega X\left( \omega \right)$$

**Fig. 1:**
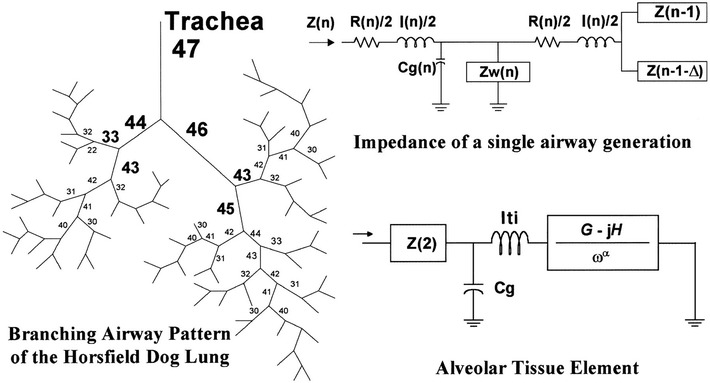
Schematic of the Horsfield lung model. Individual airways are modeled with a lumped six element model with an alveolar tissue element at the terminal units based on a given order (n) and a recursion index (Δ). The dog lung model depicted here is comprised of 47 airway orders with a defined length and diameter. Each airway consists of a resistive component (R), an inertial component (I), and as well as a term to account for shunting into gas compression in the tube (Cg) and into nonrigid airway walls (Z_w_). The viscoelastic alveolar tissue element is modeled with a tissue damping term (G) coupled to elastance (H) and an inertial tissue component (I_ti_) with a gas compression corresponding to the volume of the alveolus (Cg) (reproduced with permission from [54])

The single compartment model is readily applicable to data between 4 and 32 Hz in healthy and moderate disease but of course provides only three lumped properties averaged over the entire respiratory system with no resolution as to how airways, lung tissue or heterogeneous disease impacts them. When taking data out to higher frequencies (64–128 Hz) a variant of the six-element lumped model that was initially introduced by Dubois et al. [13] has been suggested in which airway resistance and inertance (*R*_*aw*_, *I*_*aw*_) are separated from tissue resistance, inertance, and compliance (*R*_*ti*_, *I*_*ti*_, *C*_*ti*_) by a shunt compliance to represent the compressibility of alveolar gas (*C*_*g*_) (Fig. 1) [52–54]. However, when applied to human data, this model fails as the data are insufficiently sensitive to a resonant peak associated with gas compression [55, 56].

It is now appreciated that one of the most important functional consequences of any lung disease is that it impacts the lungs heterogeneously. For example, when a system of parallel impedance pathways develop heterogeneous constriction the resulting increase in overall *R*_*L*_ and *E*_*L*_ at typical breathing frequencies is much higher than what would be predicted simply from the average decrease in all airway diameters [19, 22]. To capture the impact of disease on lung tissue and heterogeneous properties one can apply inverse models to data at lower frequencies. A commonly used model is the constant phase model from 0.1 to 4 Hz. The origins of the constant phase model relative to data derived from an alveolar capsule technique was used to explicitly measure parenchymal tissue viscoelasticity [51, 57] and partition tissue properties from airway properties within the respiratory system. In normal lungs, *R*_*aw*_ was found to remain relatively constant with increasing frequency while *R*_*ti*_ was found to decrease in a near-hyperbolic manner [58–61]. Tissue viscoelasticity is best captured with a constant phase model [62] based on a variant of the Horie and Hildebrandt model for stress-relaxation [63–65]. *Z*_*L*_ can be divided into its airway (*Z*_*aw*_) and tissue (*Z*_*ti*_) components described below [62]:

6 $$Z_{L} \left( \omega \right) = Z_{aw} \left( \omega \right) + Z_{ti} \left( \omega \right)$$

7 $$Z_{aw} \left( \omega \right) = R_{aw} \left( \omega \right) + j\omega I_{aw} \left( \omega \right)$$

8 $$Z_{ti} \left( \omega \right) = \frac{G - jH}{{\omega^{\alpha } }}$$

In which *G* is a coefficient reflecting viscous energy dissipation (also known as tissue damping) and *H* is a coefficient reflecting energy storage (also known as tissue elastance). The parameter, *α*, is related to *G* and *H* given as:

9 $$\alpha = \frac{2}{\pi }\tan^{ - 1} \left( {\frac{H}{G}} \right)$$

Constant phase refers to the phase angle between pressure and flow across the lung tissue in which the component tan^−1^(*H/G*) is constant and independent of frequency. Based on the model, the *R*_*ti*_ component of *Z*_*ti*_ can be given as:

10 $$R_{ti} (\omega ) = \frac{G}{{\omega^{\alpha } }}$$

To determine the model parameters, a nonlinear gradient search technique can be used by minimization of a performance index, Φ, for the estimation of the “goodness-of-fit” index, σ^2^, given as [18]:

11 $$\varPhi = \sum\limits_{k = 1}^{N} {\left\{ {\left[ {Re_{d} \left( k \right) - Re_{m} \left( k \right)} \right]^{ 2} + \left[ {Im_{d} \left( k \right) - Im_{m} \left( k \right)} \right]^{ 2} } \right\}}$$

12 $$\sigma^{2} = \frac{\varPhi }{2N - P}$$

where *Re*(*k*) and *Im*(*k*) denote the real and imaginary components of *Z*_*L*_ at the *kth* frequency, respectively, and the subscripts *d* and *m* denote the actual data and model predicted values, respectively. *N* is the number of frequencies, and *P* is the number of free parameters in the model. Applying the parameters of this model, *R*_*L*_ can be partitioned into its airway (i.e., central) component, *R*_*aw*_ and tissue (i.e., peripheral) components, *R*_*ti*_. Interestingly, *R*_*ti*_ has been found to constitute a substantial component of *R*_*L*_ in healthy subjects (~40%) while *R*_*aw*_ has been found to constitute a substantial component of *R*_*L*_ in asthmatic subjects (>70%) [18, 23]. The major limitation of using the constant phase model is that when airway constriction is heterogeneous it creates amplified frequency dependence in both *R*_*L*_ and in *E*_*L*_ (see “Forward modeling” section). The constant phase model has only one parameter, *G*, that can amplify frequency dependence. Hence when inversely modeling these data, *G* increases but this has nothing to do with the change in tissue properties [66]. Hence while low frequency FOT data itself is highly sensitive to the occurrence of heterogeneous constriction, inverse modeling approaches cannot distinguish heterogeneous constriction from changes in tissue viscoelasticity from these data.

### Forward modeling

A powerful approach to assess how constriction patterns and tissue properties can impact lung function is to use a forward model to predict impedances at various frequency ranges and interpret the results obtained from inverse modeling. Here, a baseline airway tree was generated from morphometric studies on the lungs by Horsfield et al. [67, 68]. Airway wall properties were incorporated which assumed that the wall consists of both soft tissue and cartilage with a wall thickness, *h*, as a function of airway generation dependent on airway radius, *r*_*c*_, and cross-sectional wall area, *WA*, described below [52, 54, 69, 70].

13 $$h = \sqrt {r_{c}^{2} + \frac{WA}{\pi }} - r_{c}$$

**Fig. 2:**
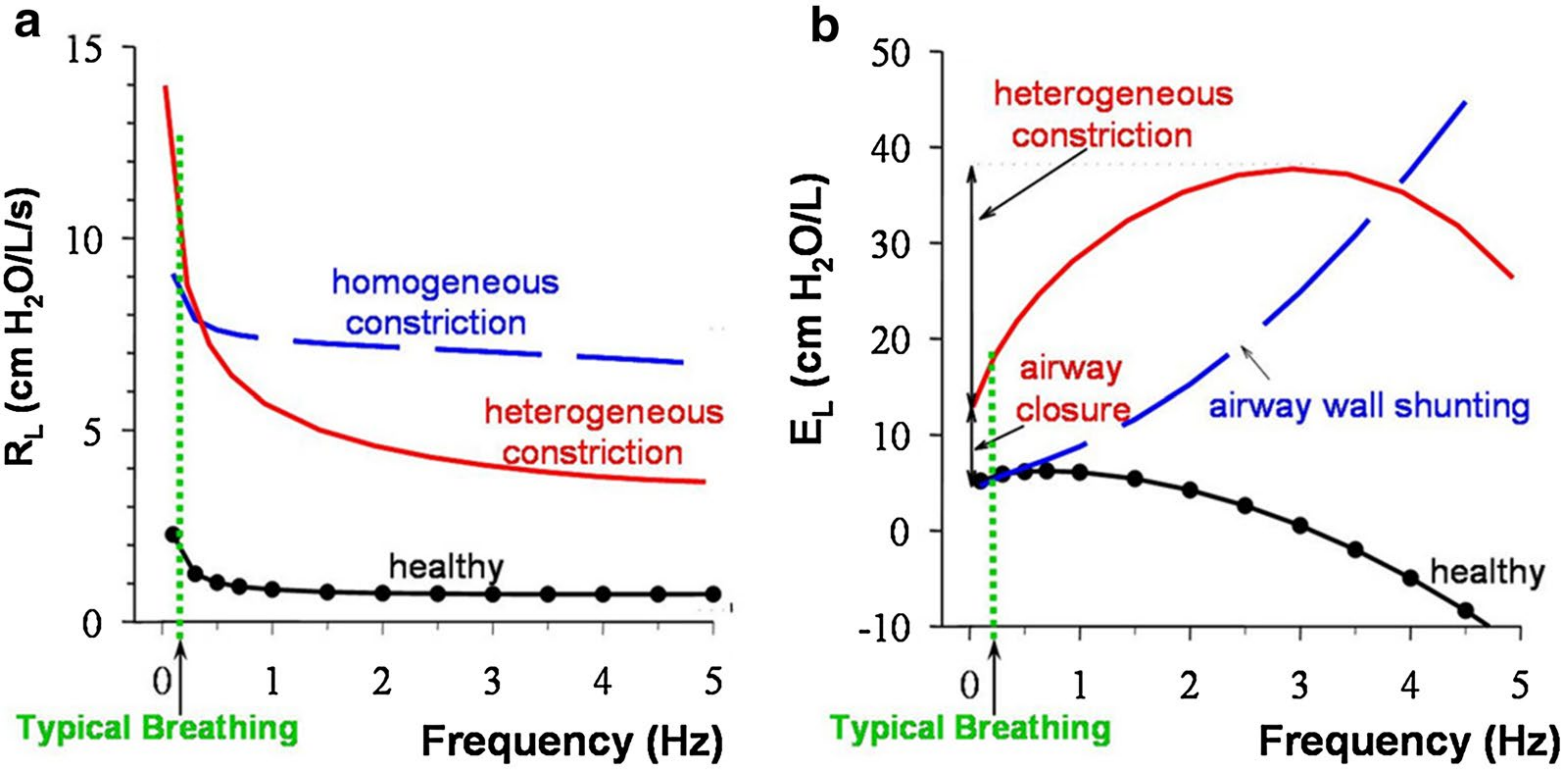
Modeled lung resistance (**a**) and lung elastance (**b**) derived from an airway tree model. Homogeneous constriction leads to a uniform elevation in R_L_ and significant shunting into the compliant central airways presenting as a progressive increase in E_L_ with frequency. Heterogeneous constriction leads to an increased frequency dependence in both R_L_ and E_L_ (reproduced with permission from [19])

Constriction was applied to peripheral airways with diameters <0.4 mm, scaled to FRC (above a Horsfield order of 6), by varying means, *μ*, and coefficients of variation, *CV*, in the reduction of airway diameters based on a Gaussian distribution. At baseline from 0.1 to 1 Hz, *R*_*L*_ exhibited a mild frequency dependent decrease followed by a plateau from 2 to 5 Hz while *E*_*L*_ exhibited a mild frequency dependent over the same frequencies followed by a decrease due to airway inertance that becomes more dominant at higher frequencies (Fig. 2) [54]. These findings were consistent with the notion of viscoelastic tissue properties residing over the lower frequency ranges.

**Fig. 3:**
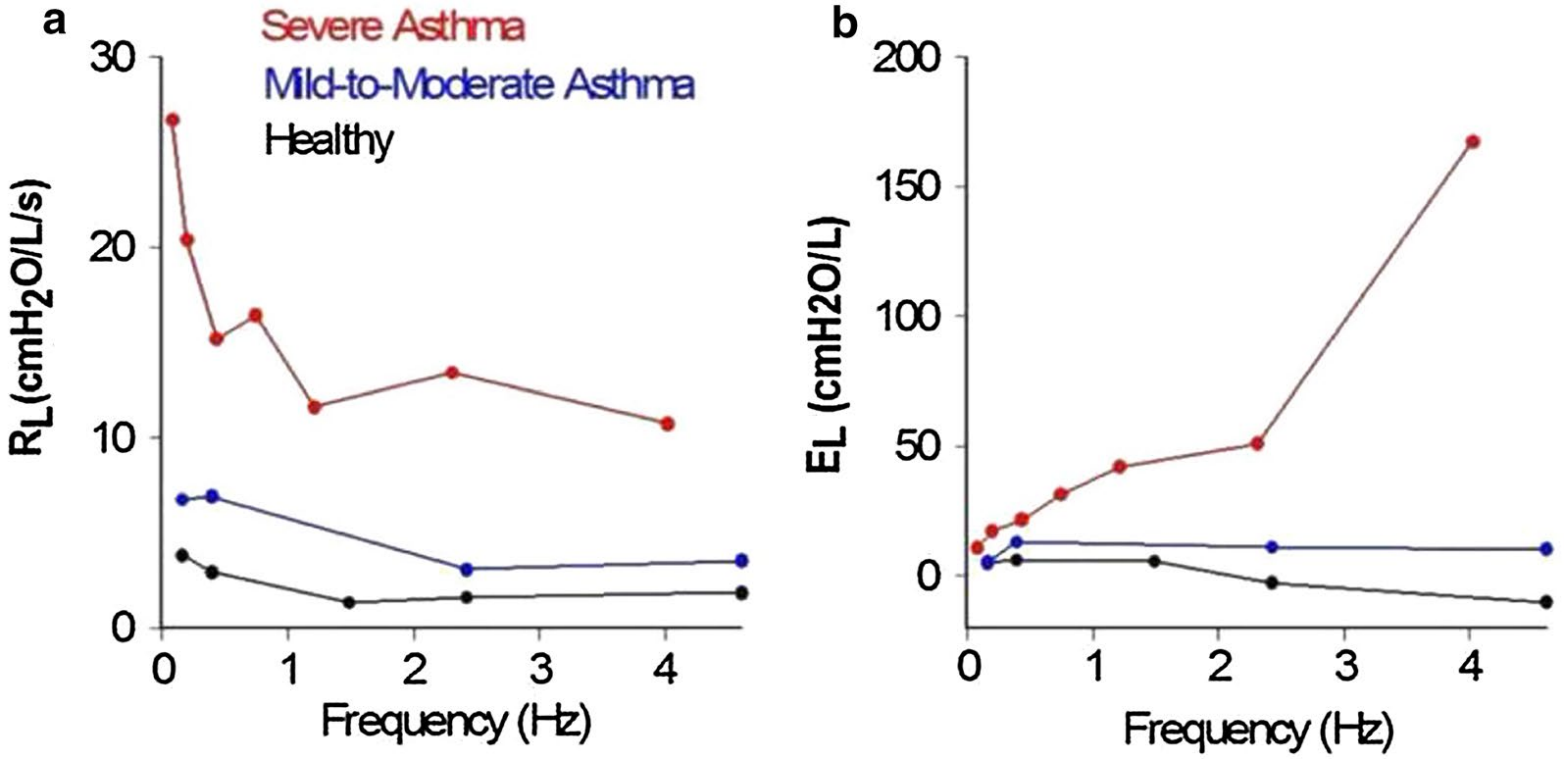
Measured lung resistance (**a**) and lung elastance (**b**) over a wide array of frequencies using an OVW technique in healthy, mild-to-moderate asthma, and severe asthma. With increasing severity of disease, there is more frequency dependence in both R_L_ and E_L_ with significant shunting at high frequencies apparent in E_L_ (adapted with permission from [19])

Homogeneous constriction (varying *μ* with minimal changes in *CV*) resulted in uniform increase in *R*_*L*_ at all frequencies without a noticeable increase in *E*_*L*_. In striking contrast, heterogeneous constriction (varying *CV* with minimal changes in *μ*) resulted in a substantial frequency dependence with a frequency dependent decrease in *R*_*L*_ and a frequency dependent increase in *E*_*L*_ mostly taking place at frequencies <2 Hz. Here, while the net increase of *R*_*L*_ at 4 Hz was far lower than during homogeneous constriction with a much higher mean reduction of airway diameter, the increase in *R*_*L*_ and *E*_*L*_ at typical breathing rates was much larger at spontaneous breathing frequencies. In other words, heterogeneous constriction serves to amplify the reduction in mechanical and ventilation lung function from that predicted by the average reduction in airway diameter across the whole tree. This same behavior is observed when expanded to a three-dimensional geometric model of the lung [41]. Moreover, when a few (~10%) of these peripheral airways are closed but distributed in a heterogeneous fashion, both *R*_*L*_ and *E*_*L*_ undergo substantial elevations with more frequency dependence when heterogeneous constriction is applied [52, 54]. In addition, there can also be a significant increase in *E*_*L*_ at higher frequencies believed to be secondary to pressures shunting across the walls of the central airway [15]. Together, these findings support the notion of heterogeneous airway closures occurring mainly at the lung periphery [54]. When translated to the measured data, the frequency dependent behavior of *R*_*L*_ and *E*_*L*_ provide rich physiological insights on mechanisms of dysfunction that may be occurring in asthmatic subjects (Fig. 3) [18, 19, 23].

## Heterogeneity of lung ventilation

By synthesizing structurally consistent forward models with data from oscillatory lung mechanics at low frequencies, we can detect that indeed airways in asthmatics constrict heterogeneously. We further show that the frequency dependence and heterogeneity seem to worsen with disease severity [19] and that, consequently, mechanical lung function rapidly declines which greatly amplifies the work of breathing. What we cannot tell from such data is explicit insight as to physically where and to what degree the ventilation distribution has been degraded. Moreover, we cannot quantify ventilation distributions from mechanical heterogeneity alone and whether the degree of ventilation degradation can lead to amplified mismatches in perfusion and eventually the ability to maintain proper blood gases.

### Multiple breath nitrogen washout

Multiple breath nitrogen (N_2_) washout (MBNW) is a non-invasive approach in the quantitative assessment of ventilation heterogeneity [71–79]. The technique allows prediction of two types of gas transport in the periphery, one that is diffusion–convection-dependent (*S*_*acin*_) and one that is convection-dependent (*S*_*cond*_), based on the pattern of N_2_ washout from the lung during tidal breathing of 100% oxygen [80]. *S*_*acin*_ and *S*_*cond*_ are calculated slopes that reflect the degree of acinar and conductive airway heterogeneity, respectively. Increases in *S*_*acin*_ and *S*_*cond*_ can be found following bronchoprovocation by methacholine in both healthy and asthmatic subjects [72, 78]. A lung clearance index (LCI) has been another parameter also used to quantify the degree of ventilation heterogeneity. Although LCI has been found to be correlated to *S*_*acin*_ and *S*_*cond*_, its utility and interpretation is still limited [79] since it is a bulk index of reduced ventilation efficiency over the entire lung and not an explicit index of heterogeneity of ventilation. Applying indices of *S*_*acin*_ and *S*_*cond*_, the degree of ventilation heterogeneity at baseline has been found to be linked to the degree of AHR, a hallmark feature of asthma, and can also be used as a predictor of asthma control [71, 73–76]. However, the technique does not maintain any spatial nor specific anatomic regional information. MBNW can only partition the lungs into two main regions: a more central conductive airway region and a more peripheral acinar region. In addition, there were questions of whether the technique can be used to detect poorly ventilated to completely non-ventilated areas of the lungs given findings from recent modeling studies [78].

### Hyperpolarized helium-3 magnetic resonance imaging

**Fig. 4:**
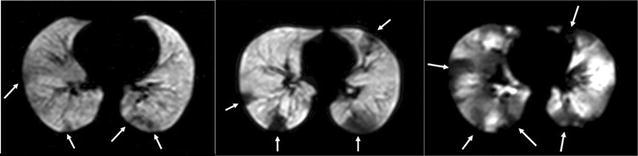
Hyperpolarized helium-3 MRI axial slice of a mild asthmatic (*left panel*), a moderate asthmatic (*middle panel*), and a severe asthmatic (*right panel*). As depicted by the *arrows*, increasing severity of disease is associated with increasing ventilation defects and ventilation heterogeneity (reproduced with permission from [28])

**Fig. 5:**
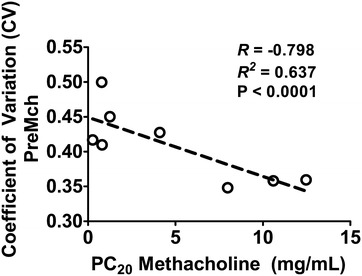
Relationship between baseline CV and AHR. Here, AHR was measured by the concentration of methacholine that elicits a 20% decrease in the subject’s FEV_1_ compared to baseline (PC_20_) dose. Lower PC_20_ doses denote higher degree of AHR. Note that there is a negative correlation between the baseline levels of ventilation heterogeneity, (i.e., the *CV*), and the PC_20_ dose in the asthmatic subjects (reproduced with permission from [32])

HP ^3^He MRI is a novel imaging modality that directly renders ventilated areas in the lungs through inhalation of a noble gas mixture. While normal healthy lungs tend to exhibit a homogenously distributed pattern of ventilation, diseased lungs, such as in asthma, tend to exhibit patchy areas of ventilation defects (very low levels of HP ^3^He) distributed heterogeneously throughout the lungs (Fig. 4) [27–37, 81]. The number and size of ventilation defects have been found to be correlated to spirometry with how the gas redistributes in the lung to be related to asthma severity [82, 83]. A substantial number (~75%) of these ventilation defects persisted or recurred at the same location, and most (~71%) did not change in size [29, 30, 80] although new ones can occur as well over time. Original analysis of these images for assessing ventilation defects were subjective at best, while more recently we have seen the emergence of quantitative approaches [28–30]. One approach has been calculating a *CV* of signal intensity within these images as a surrogate for ventilation heterogeneity [27, 32, 37]. Unsurprisingly, ventilation heterogeneity (i.e., the *CV*) was found to increase following bronchoconstriction with methacholine [27, 32, 37]. Additionally, the degree of ventilation heterogeneity was also found to be correlated with measurements of lung mechanics [32], further reinforcing the relationship with asthma severity found from prior qualitative studies which compared measurements to spirometry [28]. Moreover, concordant with previous findings using MBNW, the degree of ventilation heterogeneity at baseline was also found to be correlated to AHR (Fig. 5) [32]. This finding has very important implications: (1) whether underlying airway conditions prior to bronchoprovocation is a critical factor leading to increased AHR and (2) whether a preexisting abnormal pattern of ventilation (i.e., heterogeneity) will enhance the subsequent degradation in lung function consistent with instability mechanisms proposed through modeling studies [39, 84].

## Image functional modeling to distill specific anatomic airway origins of reduced function

To probe airway conditions linking structural changes in the lungs to measurements of mechanical function, an approach termed image functional modeling (IFM) was devised. The method employs a three-dimensional airway tree model based on a volume filling algorithm developed by Tawhai et al. [22, 27, 85]. Parameters within the model include dimensions and location of all airways inclusive of their branching angles obtained from previous morphometric studies with human airways [68, 85]. A stack-based algorithm traverses the airway tree and determines the highest branch to designate for closure to reproduce the ventilation defect in precise anatomic locations of the model that correspond to the anatomic locations of the image. Each branch within the airway tree is modeled assuming laminar flow in a compliant walled tube and hence requires a distinct flow resistance and inertance partitioned via a shunt airway wall compliance with each parameter for an airway branch a function of its length, diameter and wall material properties (Eq. 13). The terminal airways are then connected to a gas compression associated with the alveolar gas in parallel with a constant-phase tissue model all scaled to the appropriate volume. Impedance of the entire tree as well as ventilation distribution to all alveolar regions can be calculated by the appropriate series and parallel calculations also through a stack-based algorithm. All airway diameters are scaled to FRC, and the subsequent lung volume at FRC is distributed evenly among the terminal alveolar units.

**Fig. 6:**
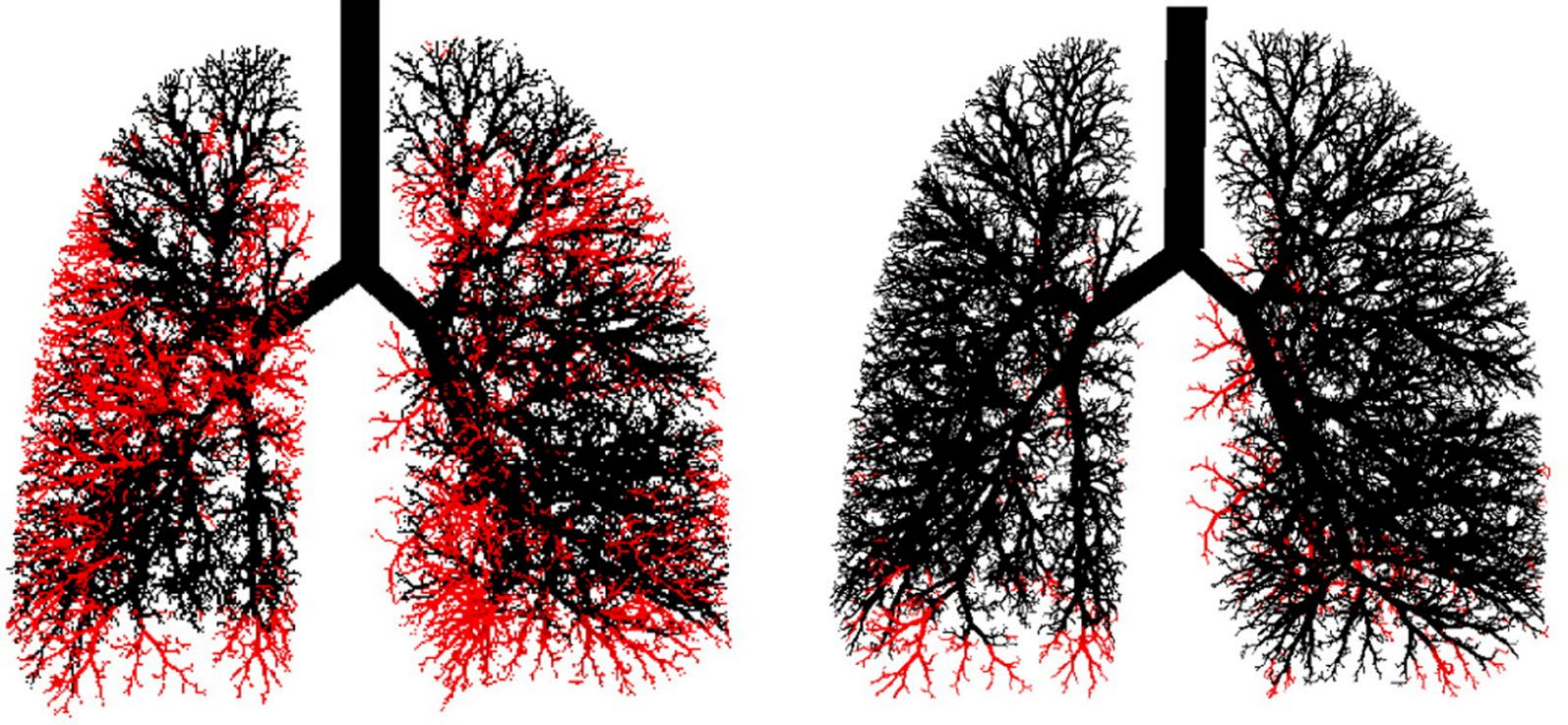
A model airway tree for an asthmatic subjects following bronchoconstriction with methacholine (*left*) and following bronchodilation with albuterol (*right*). Open ventilated airways are depicted in *black* with closed nonventilated airways depicted in *red*. Note that following bronchodilation, most, but not all, of the airways recover (reproduced with permission from [27])

**Fig. 7:**
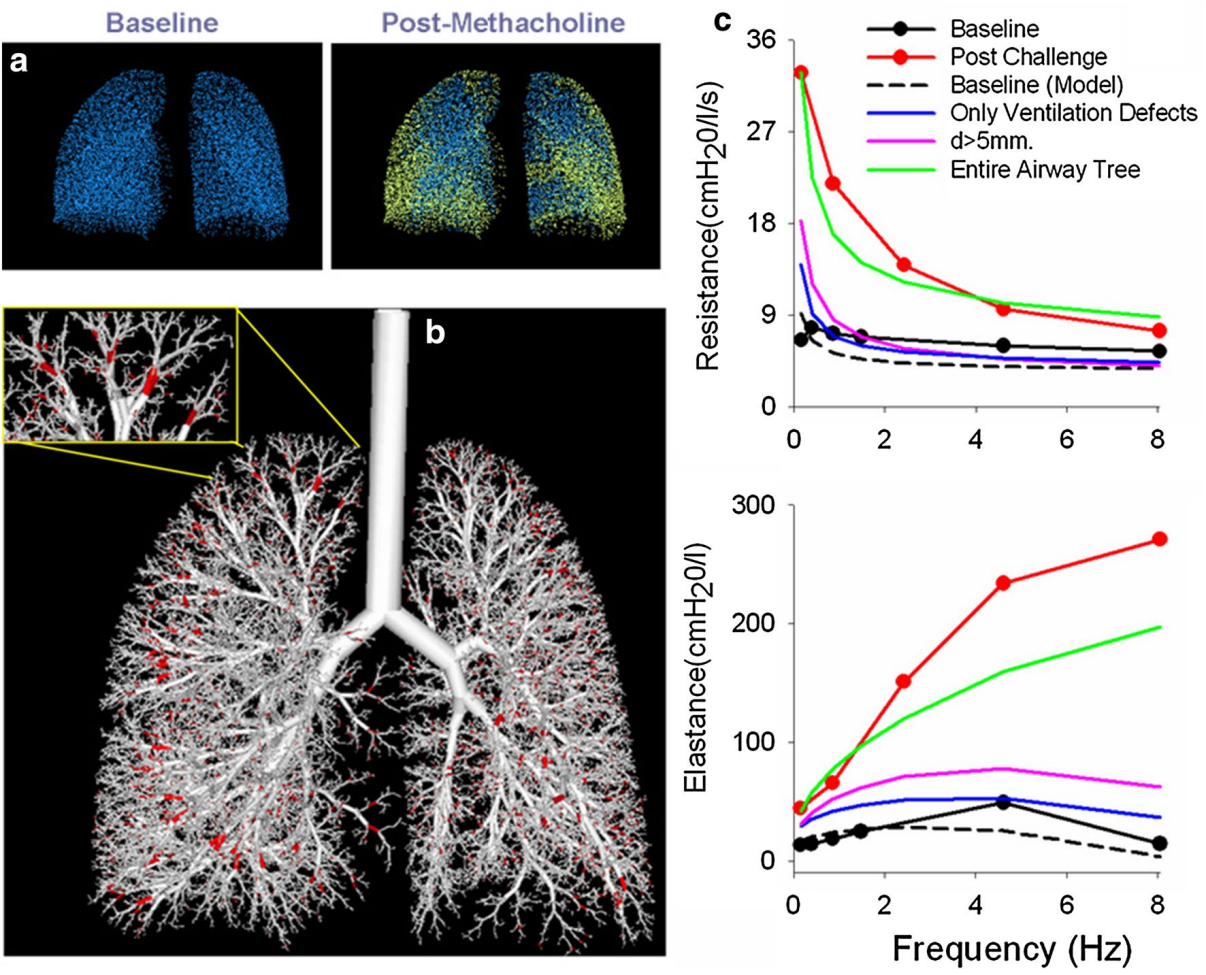
Overview of image functional modeling. **a** Terminal units are defined from the airway tree model which are subsequently mapped to the ventilation defects obtained from HP ^3^He MRI with ventilated terminal units depicted in *blue* and the nonventilated terminal units depicted in *yellow*. **b** Through a recursive algorithm, the largest airway is determined and designated for closure depicted in *red*. **c** Constriction patterns are applied to the airway tree model to best match measured R_L_ and E_L_ (reproduced with permission from [15])

One can synthesize these forward models with imaging and FOT data taken in asthmatics after inducing airway constriction by administering an airway smooth muscle agonist (i.e., methacholine). Heterogeneous constriction patterns can be imposed onto the airway tree to simulate oscillatory lung mechanics (Fig. 6) to best match measured frequency dependence of *R*_*L*_ and *E*_*L*_ determined by Eq. 11 [41, 52–54]. Simultaneously, one can overlay data from ventilation imaging of the lung obtained from PET [21, 22] and HP ^3^He MRI [27] onto the 3D anatomic airway tree and then impose on the model the appropriate anatomic locations of the sites of airway closures and/or ventilation defects to match the imaging data (Fig. 7). This is accomplished by first scaling the airway tree model to the volume defined by ventilation imaging followed by subsequent mapping of the terminal alveolar units to each ventilation defect. What is unique and ideal about the approach is that it isolates airway conditions that requires simultaneous matching of two functional measures for heterogeneity in lung mechanics and in ventilation.

It was found that for mild to moderate asthmatic subjects, one cannot match both data sets simultaneously by only constricting or closing larger airways. Doing so either caused ventilation defects in model locations that did not occur in the actual imaging data, impedance increases inconsistent with the data, or both. The only way to match both data simultaneously required heterogeneous closures of small peripheral airways (<2 mm) in the airway tree [22, 27]. The airways leading to these ventilation defects did not need to be completely closed but rather constricted by ~70% of their baseline diameters [27]. This implies that although these airways may be ventilated, they operate with a time constant such that they are functionally closed in gas exchange [27]. Moreover, severe constriction of the terminal alveolar units and airways leading to these ventilation defects were not sufficient to explain the degree of mechanical dysfunction during bronchoconstriction [21]. Although anatomically consistent airway tree models have been designed to explain ventilation defects via large airway closures, their significance and mechanical implications are still unclear [40]. It has been speculated that with more severe asthma, airway closures may occur in larger airways (≥2 mm) due to airway remodeling and inflammation. Moreover, computational modeling studies have suggested that there may indeed be some coupling between the constriction of larger airways and smaller airways leading to heterogeneous clustering of ventilation defects [84].

## Summary and conclusion

Over the past few decades substantial evidence has emerged that heterogeneous constriction, particularly in the peripheral airways, are key contributors to measures of reduced mechanical and ventilation function in lung disease [11, 86]. Previously, it was believed that due to their extensive parallel nature, these areas in the lung represented the silent zone with negligible resistive contributions to the total lung resistance. This is true for the healthy lung. However, advances in FOT and imaging have illustrated an intrinsic degree of heterogeneity that affects both large and small airways. The FOT captures the impact via the nature of a parallel tree structure to dramatically amplify the frequency dependent behavior of mechanical indices. This is accomplished in a fashion that would amplify *R*_*L*_ and *E*_*L*_ to a much greater extent due to network topology than one would predict by knowing the average diameter reduction of any single airway. Such insights become more prevalent using inverse and forward modeling schemes. More recently, imaging has evolved to enable visualization of ventilation in the lungs. Unsurprisingly, this same phenomenon of heterogeneity present in lung mechanics has also been prevalent in the pattern of ventilation and has been found to be associated with AHR that occurs in asthma. Indeed non-invasive imaging has great potential to assess the likelihood and degree of AHR (i.e., if there are baseline ventilation defects) and for tracking the efficacy of therapy to impact reactivity if it can remodel the airways to reduce baseline heterogeneity. Through combining measurements of mechanics and advances in imaging, we now have a powerful tool to explain how these structural heterogeneous changes lead to the dysfunction seen in asthma.

## Abbreviations

AHR: airway hyperresponsiveness
*Q*_*ao*_: flow at airway opening
*P*_*ao*_: pressure at airway opening
*R*_*L*_: lung resistance
*E*_*L*_: lung elastance
FOT: forced oscillation technique
HP ^3^He MRI: hyperpolarized helium-3 magnetic resonance imaging
*P*_*cw*_: pressure at the chest wall
*Z*_*tr*_: transfer impedance
*Z*_*in*_: input impedance
*Z*_*rs*_: respiratory system impedance
*Z*_*L*_: lung impedance
*ω*: frequency, in radians
*R*_*rs*_: respiratory system resistance
*X*_*rs*_: respiratory system reactance
*Z*_*L*_: lung impedance
*P*_*es*_: intraesophageal pressure
*P*_*ip*_: intrapleural pressure
*P*_*tp*_: transpulmonaty pressure
FRC: functional residual capacity
OVW: optimal ventilation waveform
*E*_*rs*_: respiratory system elastance
*I*_*rs*_: respiratory system inertance
*ω*_*0*_: resonant frequency
*R*_*aw*_: airway resistance
*I*_*aw*_: airway inertance
*R*_*ti*_: tissue resistance
*I*_*ti*_: tissue inertance
*C*_*ti*_: tissue compliance
*C*_*g*_: gas compliance
*Z*_*aw*_: airway impedance
*Z*_*ti*_: tissue impedance
*G*: coefficient of tissue damping
*H*: coefficient of tissue elastance
α*α*: constant phase parameter
*h*: wall thickness
*r*_*c*_: airway radius
*WA*: airway cross-sectional wall area
*μ*: mean
*CV*: coefficient of variation
MBNW: multiple breath nitrogen washout
*N*_*2*_: nitrogen
*S*_*acin*_: diffusion–convection-dependent gas transport
*S*_*cond*_: convection-dependent gas transport
LCI: lung clearance index
PET: positron emission tomography
IFM: image functional modeling
